# The Effects of Climate Change on the Nesting Phenology of Three Shorebird Species in the United States

**DOI:** 10.3390/ani13152459

**Published:** 2023-07-29

**Authors:** Virginia E. Abernathy, Abby Good, Autum Blanchard, Marlisa Bongiovanni, Emily Bonds, Hampton Warner, Eleni Chaknis, Gabriella Pulsifer, Faith Huntley

**Affiliations:** 1 Department of Biological Sciences, Clemson University, Clemson, SC 29631, USA; abby.kr.good@gmail.com (A.G.); blanchardas@g.cofc.edu (A.B.); marlisab53@gmail.com (M.B.); emilyhbonds@gmail.com (E.B.); hpwarne@g.clemson.edu (H.W.); elenichaknis@gmail.com (E.C.); pulsiferg27@gmail.com (G.P.); faith.e.huntley@gmail.com (F.H.); 2 GAI Consultants, Homestead, PA 15120, USA; 3 Department of Geology and Environmental Geosciences, University of Charleston, SC at the College of Charleston, Charleston, SC 29424, USA; 4 Graduate School of Education & Human Development, George Washington University; Washington, DC 20052, USA; 5 School of Medicine Greenville, University of South Carolina, Greenville, SC 29605, USA; 6 East Cooper OB/GYN, Mount Pleasant, SC 29464, USA

**Keywords:** black-necked stilt, climate change, clutch initiation date, Geographic Information System (GIS), museum collections, nesting phenology, shorebirds, Willet, Wilson’s plover

## Abstract

**Simple Summary:**

Previous studies have shown how climate change can lead some bird species to alter the timing of their breeding and migration. To understand this better in shorebirds, we investigated if the nesting times of three shorebird species in the United States have fluctuated due to increases in temperature and precipitation at their breeding sites. We found that the laying dates of our species have become earlier and temperatures at our nest sites have significantly increased over time. Precipitation did not show a clear trend over time in our study, though previous studies indicate precipitation has been increasing in the U.S. While we were unable to find evidence that precipitation affects when our species begin laying eggs, one species, the Willet, had significantly earlier laying dates as temperatures during the breeding months increased. While another species showed this same trend, it was not significant, and the third species showed later laying dates as temperatures increased. Our results demonstrate that changes in climate affect species differently, warranting further study in this field.

**Abstract:**

Previous research suggests that a frequent response of organisms to the ongoing climate crisis is the adjustment of their reproductive timing or breeding phenology. Shorebirds may be especially vulnerable to increasing temperatures and precipitation, as many are migratory and depend on coastal habitats for wintering and breeding. These particular habitats could be at risk due to changes in climate, and nesting times often depend on food availability, which is often directly influenced by temperature. We investigated if clutch initiation dates (CID) for three shorebird species in the United States have become earlier over time with increasing temperatures and precipitation. We used nest records from Cornell’s NestWatch program and various museum databases and weather station data from the National Oceanic and Atmospheric Administration. We found evidence that CIDs have become earlier over time, though this was only a significant factor for one species. While temperature in our study areas has increased significantly over time, precipitation changes were more variable and not always significantly predicted by time. We found evidence that one species may be responding to increasing temperatures by nesting earlier, but there was no support for our hypothesis that CID has changed due to changes in precipitation for any species. Results varied for each species, indicating the importance of further studies on shorebirds as the effects of climate change on their nesting phenology may not be fully realized and will likely depend on the species’ biology and distribution.

## 1. Introduction

Increasing temperatures across the globe [1] have led to observed changes in the environment that can affect migratory bird species, such as the timing of snow melt in the Arctic [2,3], the emergence of spring vegetation [4], and essential food sources for breeding adults and their young [5,6]. Migratory species may be more vulnerable to these types of changes than resident species because they rely on different wintering and breeding habitats, as well as stopover sites during migration. While some migratory bird species might be able to adjust their timing of migration and nesting based on these recent environmental changes [4,7], others may lag behind or arrive too early to the breeding grounds before conditions are optimal for nesting [8,9,10]. Any shifts or mismatches in nesting phenology with respect to the availability of critical resources, such as nesting habitat and food, could lead to decreased reproductive success and population decline for these species [3,9,11,12].

Over the last few decades, several studies have found that some bird species have begun nesting earlier (e.g., [13,14,15,16,17]), which might be a response to increasing temperatures and the earlier emergence of spring vegetation and food sources, as was shown for the Willet (*Tringa semipalmata semipalmata*) by Smith et al. [4], and for several passerine species in the U.S. by Mayor et al. [10]. In contrast, Kwon et al. [11] found that three shorebird species that breed in the Arctic have delayed clutch initiation and shortened their incubation period over two decades, potentially in response to a cooling climate trend at the start of their breeding season. Kwon et al. [3] found evidence that phenological mismatches in Arctic shorebirds were likely related to changes in temperature and timing of snowmelt, which may be one cause that has led to population decline in the species nesting in the eastern part of the breeding range. These studies suggest that changes in global temperatures can either lead to changes in the timing of reproductive behaviors in birds or increase phenological mismatches, which may have negative consequences on their populations.

Increasing temperatures can also influence other aspects of climate, such as precipitation rates. As surface temperatures increase due to increasing greenhouse gas emissions, the rate of evaporation of surface moisture increases, which can lead to greater amounts of rainfall as reviewed by [18]. Some sources indicate that the precipitation rate in the U.S. has been increasing since the 1970s [19,20]. Further research predicts rainfall events will increase dramatically in intensity yet occur at a less frequent rate due to temperature increases exceeding evaporation rates [18,21]. These changes in precipitation could have direct effects on the habitat and reproductive success of certain bird species. For example, shorebirds nesting along coastlines could be subjected to more flooding and extreme weather events, such as tropical storms or hurricanes, as well as a loss of habitat due to sea level rise [22,23,24,25,26].

In this study, we investigated the hypothesis that rising temperatures and levels of precipitation have led to earlier laying dates in migratory bird species. Specifically, we focused on three shorebird species that breed in the United States of America: the willet (family Scolopacidae, hereafter “willet”), Wilson’s plover (*Charadrius wilsonia*, family Charadriidae, hereafter “plover”), and the black-necked stilt (*Himantopus mexicanus*, family Recurvirostridae, hereafter “stilt”). These species were chosen because they all may be especially vulnerable to a warming climate [22], and none are closely related, reducing phylogenetic effects on our results. All species in our study have been found to nest in or close to vegetation [4,27,28], the emergence of which could be directly affected by spring temperatures. These species also often nest along coastlines, putting them at a higher risk of exposure to extreme weather events and changes in habitat due to factors caused by climate change [22,23,24,25,26,29]. Shorebirds also have a fixed clutch size and typically produce only one brood a year [30,31].

We used laying dates as a measure of nest phenology change and asked three primary questions: (1) Have laying dates changed over year, latitude, or longitude? (2) Has temperature and precipitation changed over year, latitude, or longitude in our study areas? (3) Does temperature or precipitation predict laying date? We predicted that laying dates will have become earlier over time due to overall increases in surface temperature [1] and increased amounts of precipitation in the U.S. [18].

## 2. Materials and Methods

### 2.1. Study Species and Sites

**Figure 1:**
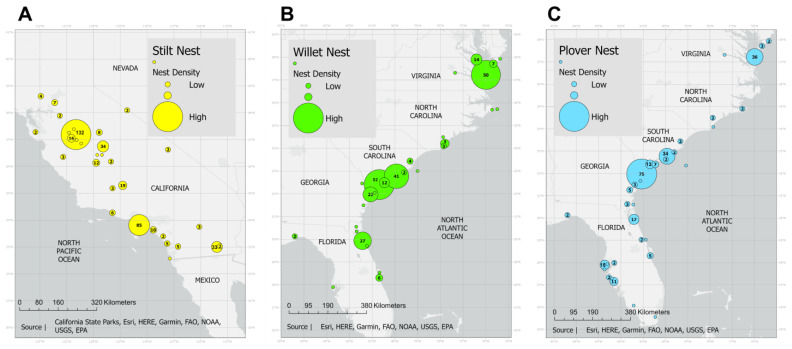
Nest locations of the stilt in California (**A**), and the willet (**B**) and plover (**C**) on the east coast of the U.S. Circle size represents the relative number of nests in an area with the actual number included inside each circle. Map created by A.G. in ArcGIS Pro 2.9 by ESRI with further edits in Photoshop by E.B.

In this study, all stilt nests were located in California, while all plover and willet nests were located along the east coast in the following states: Florida, Georgia, South Carolina, North Carolina, and Virginia (Figure 1). California is incredibly variable in climate and habitats [32]. The stilt nests were found in several types of ecoregions within this state, the top three being the Central California Valley, the Sonoran Basin and Range, and the Southern California/Northern Baja Coast (see [32] and Figure 1A). The Central California Valley is largely flat, agricultural land characterized by long, hot summers with little rainfall [32]. Likewise, the Sonoran Basin and Range is a hot, dry, desert habitat, while the Southern California/Northern Baja Coast is characterized by coastal sage scrub and chaparral with both low-lying areas by the coast and small hills. Many of the nests in the eastern states were found within a region called the North American Coastal Plain (NACP) (see [33] and Figure 1B,C). This region borders the Atlantic Ocean and consists of habitats such as closed forests, pine savannas, grasslands, and wetlands, including swamps, marshes, and bogs [29,33]. Several factors influenced by climate change, including rising sea level, increased episodes of flooding or drought, hurricanes that are more intense, and increase levels of saline in ground and surface waters, are threatening the habitats in this region [29].

Stilts have an incubation period of around 25–26 days and lay an average of four eggs per clutch over 4–5 days [34]. In our study, we assumed that stilts laid one egg each day with an incubation period of 25 days based on data collected in the Tulare Basin (average and median incubation period was 25 days, n = 43) [34], which is geographically close to the majority of the nests used in this study (Figure 1A; Supplementary Table S1). The timing of incubation for stilts appears to vary depending on ambient temperatures, but we assumed that stilts began incubating after laying the final egg. Plovers typically lay three eggs per clutch over 4–6 days. We assumed an average of five days for the laying period for a clutch of three eggs [35]. Regular incubation begins after the last egg is laid and also appears to be around 25 days, though this is based on limited observations. Willets typically lay four eggs per clutch, usually over six days. We assumed incubation began after the last egg was laid, but this has not been well-studied [36]. The incubation period is around 25–26 days, with an average of 25.2 days or a median of 25.5 days. Based on this, we assumed an incubation period of 26 days (Table 1 summarizes the metrics used to help us estimate laying date).

### 2.2. Estimating Clutch Initiation Dates

**Figure 2:**
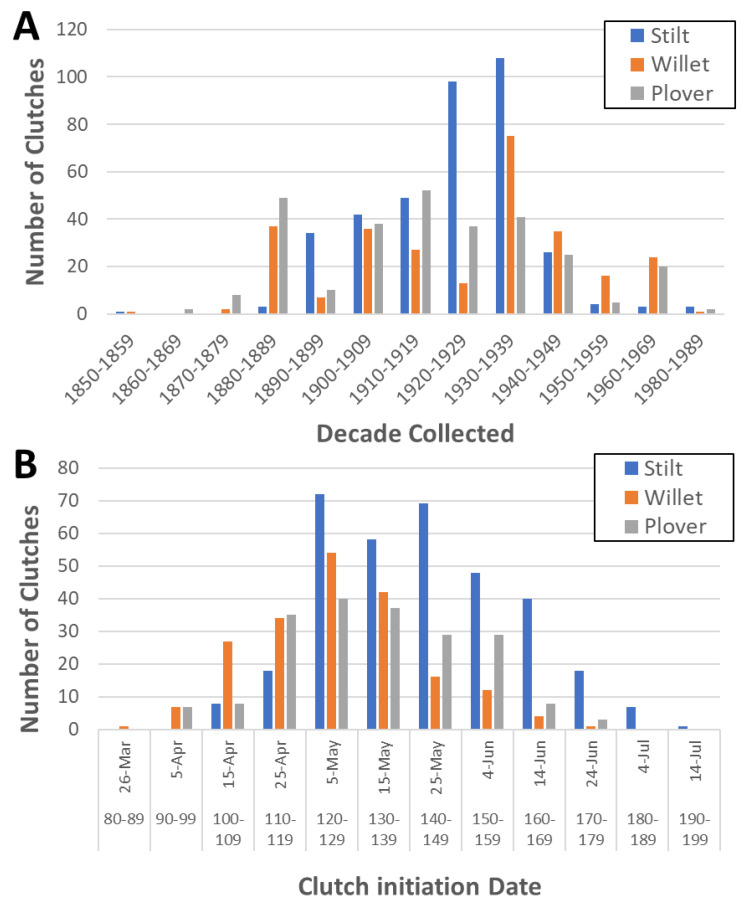
Histograms showing (**A**) the distribution of nests of each species collected from 1852 to 1989 for all 944 nests (stilt = 412, willet = 287, plover = 295) and (**B**) the timing of clutch initiation during the breeding season for each species using only nests where clutch initiation date could be reasonably estimated (total nests = 733, stilt = 339, willet = 198, plover = 196). In (**B**), the *x*-axis shows calendar dates above with the corresponding Julian date below.

To determine if the laying date or clutch initiation date (hereafter referred to as “CID”) for the willet, plover, and stilt have changed over time and with changing climate, we collected nesting information for these species from 1852 to 1983 using several museum databases, as well as nest observation cards from 1966 to 1989 provided by the Cornell NestWatch program, giving us information on a total of 994 nests (Table 2 and Figure 2A). The database website Arctos (https://arctosdb.org, accessed in October 2021) was used to retrieve records from some smaller museums (Table 2). Once CIDs were estimated for all clutches, they were converted into a Julian calendar date for use in the statistical analyses (Figure 2B and Supplementary Table S1).

From museum records, we were usually able to estimate CID using the clutch size and the collector’s description of incubation stage at the time the clutch was collected, following similar methods to McNair [43] (see also [44]). If a collector wrote down a specific number of days or a range of days in which they thought the eggs had been incubated upon collection, we used that number (or the median of the range rounded up to the nearest whole number) to estimate the length of incubation time at collection. However, many collectors described the incubation stage in categorical terms (e.g., about 1/2 incubated, advanced, etc.). Different collectors also used different terms that likely had similar meanings. For consistency, we combined similar incubation stage terms collectors used into our own incubation stage categories (e.g., fresh, slight incubation, 25% incubation, etc.) that were then translated into a specific number of days based on the species’ known incubation period (Table 1 and Table 3). Clutch size was used to determine the laying period based on what is currently known about each species’ laying behavior (Table 1). We assumed stilts laid one egg per day, so their laying period equaled their clutch size (for a clutch of four eggs, the laying period would be four days) [34]. We assumed the plover and willet laying periods were always the clutch size plus two days (for a clutch of four eggs, the laying period would be six days) [35,36]. We assumed all birds began incubating on the final day of the laying period. To account for this, we added the laying period-1 to the estimated incubation period. This total was the number of days we counted backward from the date the clutch was collected to reach an estimated CID (Supplementary Table S1) (see also [43]).

If a clutch size was two or more less than a typical full clutch, and if no incubation stage term was given by the collector (or if incubation stage was listed as “unknown”), we assumed the bird was still in the laying period, so the incubation period was 0. For example, according to Robinson et al. [34], a typical clutch size for the stilt is four. Any nests with 1–2 eggs listed as the clutch size were treated as if they were still in the laying period when they were collected if no incubation stage was recorded. Thus, a stilt nest with one egg as the clutch size collected on 4 May was given an estimated CID of 4 May. A stilt nest with two eggs as the clutch size collected on 4 May was given an estimated CID of 3 May. If the incubation stage was not recorded or labeled “unknown” and the clutch appeared to be completed upon collection, we did not attempt to estimate CID and these nests were not included in analyses where an estimation of CID was required (Supplementary Table S1).

NestWatch nest observation cards had to be treated differently from the museum records, as most of these included observations of an active nest, and observers typically did not collect and remove egg contents for long-term storage. Therefore, they could not determine what incubation stage the nest was in upon discovery simply by looking at the eggs. We used the observer’s description of the bird’s behavior and changes in nest contents over a few days to estimate CID. For example, if an observer recorded one egg on day 1 for a stilt nest, and two eggs on day 2, we assumed the nest was found during the laying period and day 1 was the CID. If an observer said the egg was pipping, but the actual hatching date was not recorded, we assumed hatching occurred the following day and counted backward from that date based on the typical incubation time and laying period for that species (incorporating clutch size as stated before). If the observer recorded that chicks were still wet, we assumed hatching occurred that day. If hatching occurred between visits within a 1–5 day range, we always assumed it occurred on the median day of the range (always rounding up to the nearest whole number). If the observer estimated the age of the young to be a range of days, we always used the median age of the range, rounding up to the nearest whole number. We did not include any clutches for the following situations:

1. Only one visit was recorded, making it impossible to know the true stage of the nest;
2. The nest was only found during incubation, with no indication of when the eggs were laid or when they started hatching;
3. Hatching occurred between visits with more than a 5-day range with no age estimate of the young;
4. Only young were observed, but there was no clear age estimate, or the number of young seen was less than the typical clutch size for that species;
5. One plover nest was not used because it may have been a later nesting attempt of the same pair that had a failed nest earlier that year in that area.

### 2.3. Nest Location and Climate Data

If latitude and longitude were provided by the collector or observer, these were considered to be the true coordinates of the nest. If only a location description was provided, we estimated latitude and longitude coordinates from the most specific locality recorded using the website epsg.io from MapTiler Team [45]. The coordinates were uploaded as a layer into ArcGIS Pro 2.9 by ESRI (Figure 1). We downloaded a list of weather stations, along with their location coordinates, for each state within our study from the National Oceanic and Atmospheric Administration (NOAA), which was also uploaded as a layer into ArcGIS. In ArcGIS, we used a tool called “Generate Near Table” to produce a list of the ten closest weather stations to each clutch. The global monthly climate summaries for each of these stations from 1851–2022 were downloaded from NOAA. From this, we used the average precipitation (cm) and temperature (Celsius) during the first four months of the breeding season for our species (March–June). We excluded July as only stilts initiated nests during the first-half of this month (Figure 2B). Unfortunately, not all stations had both precipitation and temperature data for all years the station was active, and most stations were only active a portion of the years from which we sampled data. In total, we retrieved climate data for 671 weather stations with 239 from California close to the stilt nests and 432 in the east (Florida, Georgia, North Carolina, South Carolina, Virginia) close to the plover and willet nests (Supplementary File S1).

### 2.4. Have CIDs Changed Based on Year, Latitude or Longitude for Our Three Species?

To answer this question, we ran a full linear regression model (LM) to see if CIDs for all species combined have changed over time or space. Our predictor variables included the year of the nest, nest latitude, nest longitude, species, and all interactions between species and the other predictor variables. We used a total of 733 nests in this analysis where CID and nest coordinates were able to be reasonably estimated or had been provided (stilt = 339, willet = 198, plover = 196). A second analysis was run with each species separately, allowing us to understand which factors were the most important for each species. Similar methods were used by Torti and Dunn [16].

### 2.5. Has Temperature and Precipitation Changed over Year, Latitude or Longitude in Our Study Areas?

In this analysis, we treated each weather station as a separate data point and controlled for station ID since most had been sampled multiple times over many years (Supplementary File S1). Torti and Dunn [16] separated their temperature data into four regions based on latitude (latitudes greater than vs. less than 40 degrees) and longitude (longitudes greater than vs. less than 110 degrees). In our study, all stilt nests had longitudes greater than 110 degrees, while all plover and willet nests had longitudes less than 110 degrees (Figure 1). Additionally, all nest latitudes were below 40 degrees. Based on this, we separated temperature and precipitation data into eastern and western regions and ran mixed linear models (MLM) with station ID as a random effect and year, station latitude, and station longitude as fixed effects. The response variable was either east temperature, west temperature, east precipitation, or west precipitation for each month of the main part of the breeding season (March–June). In total, we ran 16 MLMs.

### 2.6. Does Temperature or Precipitation Predict CID?

For this analysis, we used similar methods described by Torti and Dunn [16]. We estimated the temperature and precipitation at a nest for each of the first four months of the breeding season by averaging the average monthly temperature and precipitation of the ten closest weather stations to a given nest that were active during the same year the nest was active. However, in many cases, there may have been fewer than ten stations (or no stations) active during a particular year a nest was active. Therefore, our sample size for this analysis was much smaller and, in some years, may have only included data from a single weather station for a particular nest (Supplementary File S2). Next, we determined which average monthly temperature and precipitation best predicted CID for each of our three species [16]. We performed LM analyses for each species separately, using CID as our response variable and average monthly temperature or precipitation as the predictor variable for March, April, May, and June, giving us a total of four LMs for temperature for each species and four LMs for precipitation for each species. The best model was considered to be the one with the highest R^2^.

For all analyses, any interactions or individual predictor variables that were not significant (*p* > 0.05) were removed in a stepwise fashion (with the most insignificant factors being removed first) from the final model that is reported. When comparing models in the first and third analyses, we used either R^2^ (when only one predictor variable was included) or adjusted R^2^ (when more than one predictor variable was included) to determine which model was best. We considered the best model to be the one with the highest R^2^ or adjusted R^2^. In the second analysis, we report the models with the lowest Akaike Information Criterion (AIC) score because in several of these models, the adjusted R^2^ was minimally lower when some significant effects were removed, making it more difficult to interpret. In some cases, the best model may have included both significant and insignificant effects, though this was rare. All statistical tests in this study were performed in JMP Pro 16.0.0 with an alpha of 0.05.

## 3. Results

According to our estimated CIDs, willets began nesting first, with the earliest CID being on 24 March (83 Julian date) and the latest being on 23 June (174 Julian date) (Figure 2B). The average willet CID was 5 May (125.3 Julian date). Plovers began nesting as early as 3 April (93 Julian date), with the latest nest initiated on 21 June (172 Julian date) and the average CID on 12 May (132.5 Julian date). Stilt CIDs ranged from 12 April (102 Julian date) to 11 July (192 Julian date), with an average date of 22 May (142.5 Julian date).

### 3.1. Have CIDs Changed Based on Year, Latitude or Longitude for Our Three Species?

**Figure 3:**
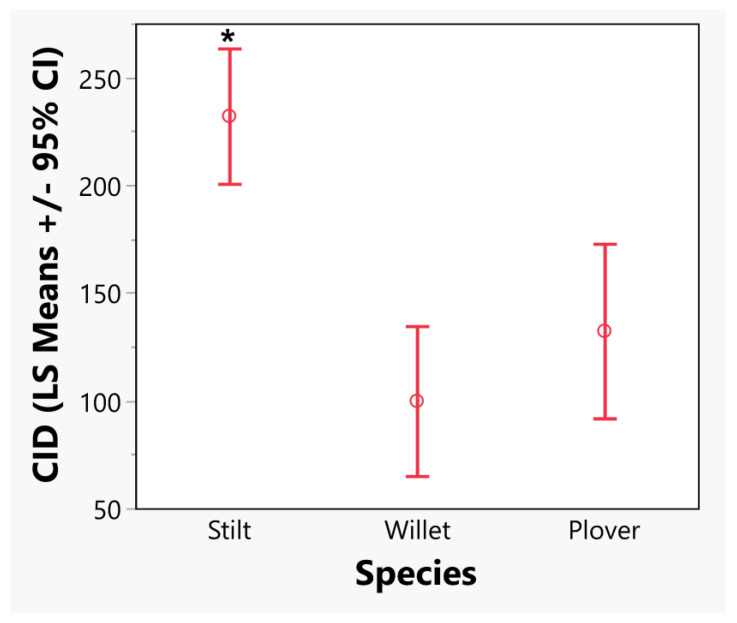
Least square means of clutch initiation dates (CID) ± 95% CI for all three species. An asterisk (*) indicates a significant difference between groups (LM: Tukey HSD, *p* < 0.05). Total nests = 733, stilt = 339, willet = 198, and plover = 196 for nests from 1852–1989.

**Figure 4:**
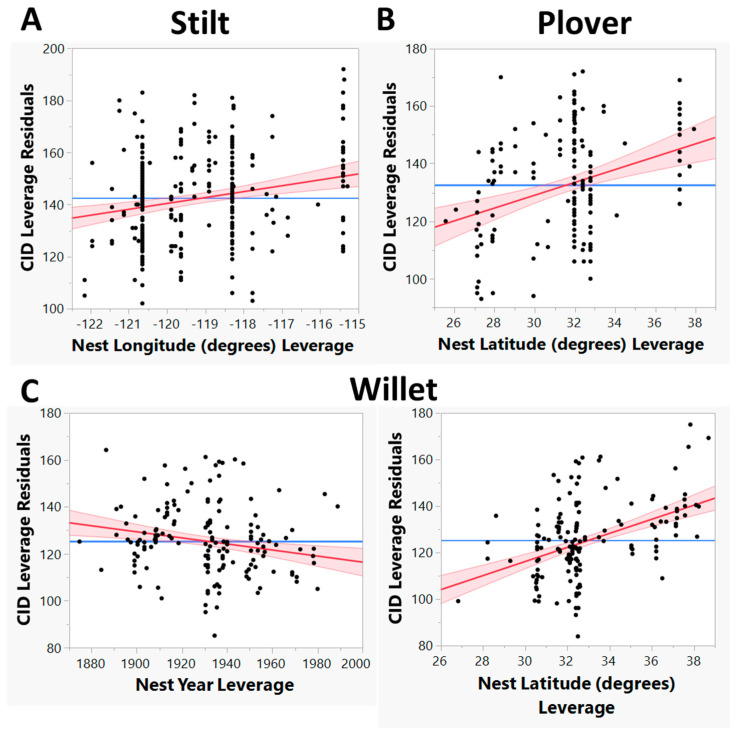
Leverage plots showing the relationship between clutch initiation dates (CID) and nest year, nest latitude, and nest longitude for the stilt (**A**), plover (**B**), and willet (**C**) in a linear model. Results are reported for the best linear model for each species which only included significant fixed effects. The red solid line is a least squares line of best fit surrounded by 95% confidence bands. The blue horizontal line represents the hypothesis that the effect in question has zero influence on CID. Stilt nests = 339, willet nests = 198, and plover nests = 196 for nests from 1852–1989.

In the model where all species were combined, all predictor variables were significant, as was the interaction between species and nest longitude (LM, adjusted R^2^ = 0.24, F_7, 725_ = 33.9, *p* < 0.001; Table 4). Stilts began nesting later than plovers and willets and were the only species in our study to start nests in July (Figure 2B). Though the majority of nests for all species were started in May, stilts had significantly later CIDs, on average, than the other two species (LM: Tukey HSD, *p* < 0.05; Figure 3). All species showed later CIDs as latitude increased, likely due to the later arrival of spring and warmer temperatures in more northern latitudes (Latitude: estimate = 1.75, *p* = 0.008). Species showed different trends in their CIDs with relation to longitude, which was explored more in the second analysis where separate LMs were run for each species. Overall, species’ laying dates have become earlier over time (Year: estimate = −0.09, *p* = 0.003), but year was not the most important predictor of CID for all species (Figure 4).

In the LM where only stilt nests were included (n = 339), the only significant predictor of stilt CID was longitude (LM: adjusted R^2^ = 0.04, F_1, 337_ = 16.5, *p* < 0.001; Longitude: estimate = 2.26, *p* < 0.001). The nests that were further inland (higher longitude values) had later CIDs than nests closer to the coast. However, there was a lot of variation in this dataset, with many nests being found at the same longitudes but showing a wide range of CIDs (Figure 4A). Plover CIDs (n = 196) were only significantly affected by latitude (LM: adjusted R^2^ = 0.10, F_1, 194_ = 22.01, *p* < 0.001; Latitude: estimate = 2.22, *p* < 0.001; Figure 4B), becoming later at more northern latitudes. Willet CIDs (n = 198) were significantly affected by year, becoming earlier over time (LM: adjusted R^2^ = 0.28, F_2, 195_ = 39.9, *p* < 0.001; Year: estimate = −0.13, *p* = 0.002), and latitude, becoming later at more northern latitudes (Latitude: estimate = 3.03, *p* < 0.001; Figure 4C).

### 3.2. Has Temperature and Precipitation Changed over Year, Latitude, or Longitude in Our Study Areas?

For this analysis, we only report results from the best models with the lowest AIC (Table 5). In our study areas, average monthly temperature has increased significantly over time for all four months (March-June) in both the west (California) and east, and these models all showed high adjusted R^2^ values (MLM: adjusted R^2^ = 0.69–0.92, *p* < 0.03 for all models; Table 5(A)). In the east, and in the west during March, temperature significantly decreased as latitude increased (colder temperatures occurred at more northern latitudes; *p* < 0.001 for all models). In June in the west, temperature increased significantly at more northern latitudes (*p* = 0.008). When longitude had a significant effect on temperature, we found for both the east and the west, temperature significantly increased further from the coast (longitude and temperature showed a positive relationship in the west but a negative relationship in the east, *p* < 0.002 for all models). In our dataset, longitude values are negative because all are in the western hemisphere. Therefore, larger values in the west indicate more inland locations, while larger values in the east indicate locations on the coast (Figure 1).

All precipitation models showed much lower adjusted R^2^ values (0.14–0.28 for the west and 0.07–0.20 for the east; Table 5(B)), which may indicate precipitation is more variable than temperature or may not be as predictable using a linear model over time or space. In the west, the year never had a significant effect on precipitation. In the east, year was always a significant effect (*p* < 0.001); however, the relationship between year and precipitation differed depending on the month. The best model for predicting precipitation changes in the west was for March (MLM: adjusted R^2^ = 0.28), which showed a significant decrease in precipitation at more northern latitudes (*p* = 0.006) and more inland longitudes (*p* < 0.001), but this relationship was reversed in May and June. The best model in the east was for June (MLM: adjusted R^2^ = 0.20), which showed an increase in precipitation over time (*p* < 0.001) and a decrease in precipitation at more northern latitudes (*p* < 0.001).

### 3.3. Question 3: Does Temperature or Precipitation Predict CID?

**Figure 5:**
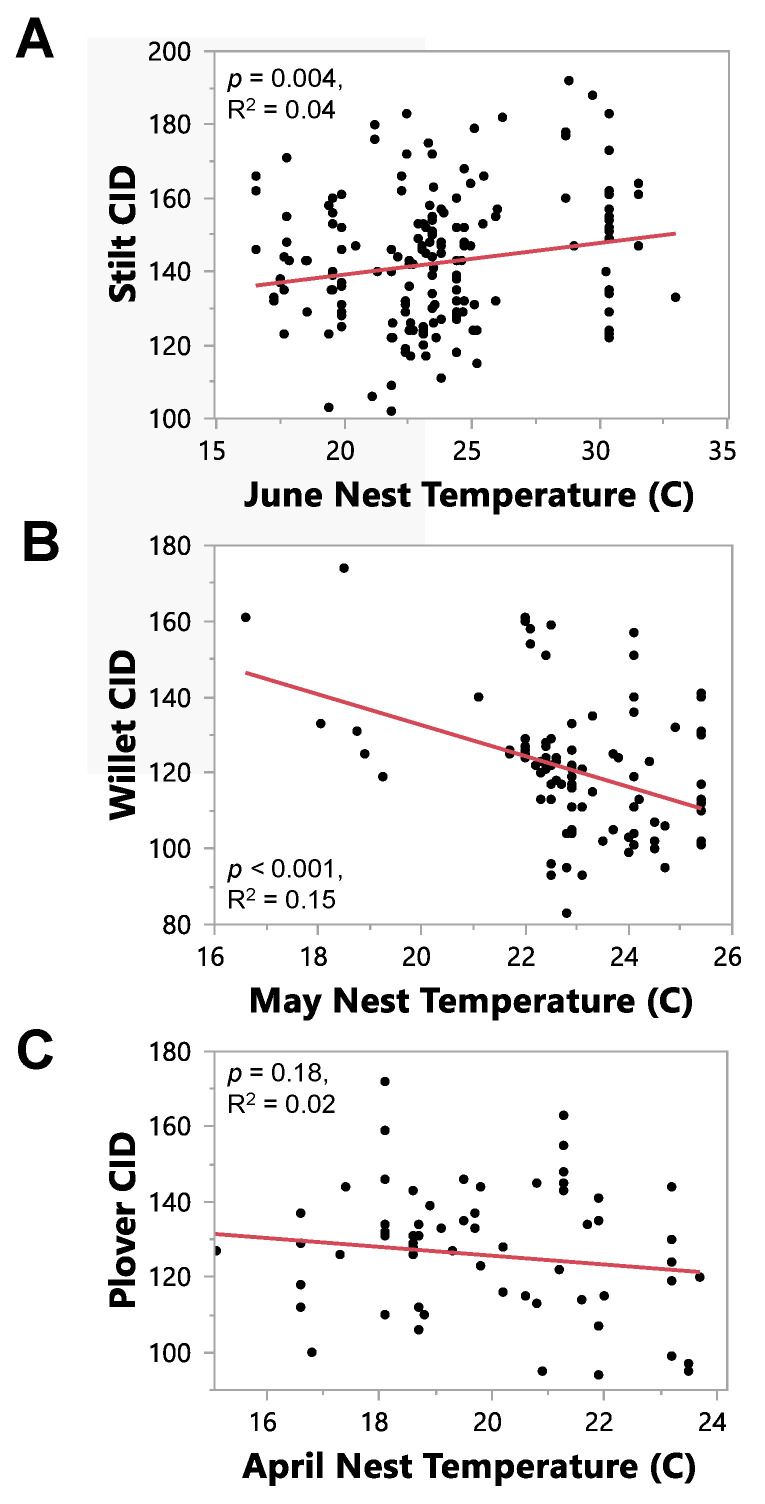
Relationship between clutch initiation date (CID) and nest temperature for the month that best predicted CID for the stilt (**A**), n = 221, years from 1896–1975; willet (**B**), n = 112, years from 1896–1989; and plover (**C**), n = 79, years from 1897–1970. The *p*-value and R^2^ values have been provided on the graph for each model.

Each species had different results for the temperature analysis (Table 6). For stilts, CIDs always became later as temperature increased (positive relationship), which was contrary to our prediction. The average June temperature best predicted CID for stilts, and this was a significant effect (LM: R^2^ = 0.04, F_1, 219_ = 8.52, estimate = 0.86, *p* = 0.004; Figure 5A). The average May temperature was also a significant predictor of stilt CID (*p* = 0.03). For willets, CIDs always became earlier as temperature increased (negative relationship), and temperature was always a significant effect for each month (*p* < 0.005 for all models), as predicted (Table 6(A)). The average May temperature best predicted CID for willets (LM: R^2^ = 0.15, F_1, 110_ = 20.11, estimate = −4.07, *p* < 0.001; Figure 5B). Temperature was never a significant predictor of plover CID for any month (Table 6(A)), but the average April temperature was the best predictor (LM: R^2^ = 0.02, F_1, 77_ = 1.83, estimate = −1.17, *p* = 0.18; Figure 5C). Plover CIDs became earlier with increasing temperature in March and April, but later with increasing temperature in May and June.

Precipitation never significantly predicted CID for any species for any month of the breeding period (LM: R^2^ = 0–0.03 and *p* > 0.07 for all models; Table 6(B)). Still, out of the four breeding months, March precipitation was the best predictor of stilt CID (LM: R^2^ = 0.01, F_1, 221_ = 3.12, estimate = −0.07, *p* = 0.08), June precipitation was the best predictor of willet CID (LM: R^2^ = 0.02, F_1, 125_ = 2.35, estimate = −0.03, *p* = 0.13), and May was the best predictor of plover CID (LM: R^2^ = 0.03, F_1, 96_ = 2.75, estimate = −0.05, *p* = 0.10). Even though precipitation was not a significant effect, CID became earlier as precipitation increased (negative relationship) for the best models for each species, which matched our predictions.

## 4. Discussion

### 4.1. Main Findings Regarding Temperature

We found partial support for our hypothesis that increasing temperatures have caused shorebird clutch initiation dates to become earlier over time. While our first model showed that CIDs became earlier when all species were combined, year was not a significant predictor of CID for the stilt or the plover when the models were run with each species separately. However, willet CIDs have become significantly earlier over time (Figure 4C) and with increasing temperatures (Figure 5B). Even though temperature was not a significant predictor of CID for plovers, the trend was in the predicted direction (Figure 5C). Unexpectedly, stilt CIDs became significantly later with increasing temperatures (Figure 5A).

Temperatures have been increasing in our study areas over time (Table 5(A)); thus, we did find support that the timing of nest initiation for willets has likely become earlier over time, due, at least in part, to increasing temperatures. These results for the willet match well with those found by Smith et al. [4], who investigated factors affecting the timing of breeding events in this same species. In their study, Smith et al. [4] found temperatures at their sites from Georgia to Maine had increased over time, leading to earlier springs, and that spring temperature was a strong predictor of willet nest initiation, along with salt marsh biomass. However, they also noted that the correlation between temperature and nest initiation was strongest in the more northern latitudes (above 36°). This pattern makes sense as locations at higher latitudes have been warming more quickly than more southern locations [46] (p. 688), [47]. A study on multiple types of animal and plant species from across the globe demonstrated that species at latitudes above 50° have exhibited stronger phenological shifts compared to those below 50° [48] (see also [49]). All of the nests in our study were below 40°. This might explain why our models looking at how well the temperature in our areas predicted CIDs had lower R^2^ values than what Smith et al. [4] found, or were insignificant in the case of the plover.

### 4.2. Main Findings Regarding Precipitation

Our hypothesis that increasing precipitation has caused shorebird CIDs to become earlier over time was unsupported. We found no evidence that precipitation was changing over time around our western nests, and whether it was increasing or decreasing over time near the eastern nests depended on the month (Table 5(B)). We found no evidence that precipitation changes could reliably predict changes in CID for any of our species (Table 6(B)). Although the best models showed the predicted trend of CIDs becoming earlier as precipitation increased, this was never significant, and R^2^ values in all models were low (0–0.03). Studies have indicated that obvious increases in precipitation in the U.S. only recently started occurring (since the 1970s) [19,20,50]. Only 66 out of 994 of the nest records we found were active from 1970 onwards, and even fewer of these nests were used in the final analysis comparing CID to precipitation near nests. This is one possible explanation for why we were unable to detect any significant trends using precipitation data. Additionally, it may be harder to use precipitation data to predict CID for birds simply because changes in precipitation, unlike changes in surface temperatures, appear to be more variable across locations due to many factors [18]. The average monthly amount of precipitation may not be as important a predictor of changes in nest phenology as changes in the frequency, duration, and intensity of rainfall events [18,51]. Further, precipitation changes in different parts of the country may be different; Trenberth [52] found rainfall occurred for 30% of an hour in the Northwest, butonly occurred 2% of an hour in California, where all of our western nests were located.

In the west, latitude and longitude significantly predicted precipitation, and the two best models showed a decrease in precipitation at more inland locations in March and April (Table 5(B)). This was interesting because stilt laying dates became later with increasing temperatures and at locations further inland (which also had warmer temperatures than locations on the coast). Stilts also did not begin nesting, in general, until mid-April. This could suggest that stilts breeding further from the coast may be delayed by higher temperatures and lower amounts of precipitation. Multiple studies on other species in North America and Europe indicate the importance of successful nesting on food availability (e.g., [4,5,6]). As stilts largely forage on macroinvertebrates in wetlands [34], both temperature and precipitation could affect their food supply. However, we could not find previous studies that specifically focused on factors influencing the timing of stilt nesting behavior. One study found that weather patterns, such as air temperature, precipitation, and storms, all seemed to influence nest initiation in stilts at a constructed wetland, but the authors did not provide evidence for this claim in the form of data or statistical analysis [53].

### 4.3. Different Responses among Species

Similar to our results, multiple studies in North America and Europe, even those that investigated many more species and nests over wider geographic areas, have found species-specific differences in response to climate changes (e.g., [7,10,13,14,16,17]). While both of our eastern species showed a trend towards earlier CID as temperatures increased, the western species showed the opposite trend. Additionally, temperatures and precipitation averages in different months predicted CID differently for each species. Mayor et al. [10] also found regional differences in passerine arrival dates in western forests vs. eastern forests and cautioned against applying patterns found in one species or one region to other species or regions not included in the study. Differences in how species respond to changes in climate can also occur at more local scales [7]. Species-specific factors, including migration distance [6,22], reproductive strategy [7], and trophic level [14], can also account for mixed responses to climate change. Additionally, changes in temperature, precipitation, or other factors related to climate change on wintering grounds or stopover sites used during migration may affect nesting phenology in different ways depending on the region and species [54]. Thus, it is imperative that more studies on various species across wide geographic ranges and time be conducted to increase our understanding of how individual species in specific regions have been affected by climate change and how flexible these species are at responding and adapting to these rapid changes in their environments. This is especially important for our study species, as Galbraith et al. [22] indicated that all three shorebird species are more vulnerable to extinction—in part due to climate change—than currently considered based on the U.S. Shorebird Conservation Plan Risk Categories. In particular, the plover was listed as “critical” by Galbraith et al. [22], the highest risk category (see also [55,56]), yet this species has been sorely understudied [27].

### 4.4. The Use of Museum Records to Study Nest Phenology

As a final point, we encourage future studies to increase their use of museum egg records to investigate nesting phenological changes in avian species (see also [17]). Museum collections are a valuable resource for scientific research due to their worldwide sampling across temporal, geographical, and taxonomic species, and museum records are typically freely available and easily accessible online [57,58]. Broad sampling can be impossible to collect while performing fieldwork, and long-term data collection is vital for phenological studies. However, the use of museum collections for phenological investigations has been criticized in the past for a possible lack of accuracy in the data from the collectors and collection biases [59]. Collection bias may lead to over-representation of certain egg traits, such as color and size [60], preference for parasitized clutches, and for clutches laid much earlier in the season than the natural variation seen in the environment [61]. However, concern about these biases may be exaggerated, as accuracy issues with a particular collector or collection are often obvious and can be accounted for if used with caution [58,62]. Further, while the information on a clutch card may be too broad or missing data, this and other studies [17,43,44,62] have demonstrated ways to reasonably estimate clutch initiation dates from the data recorded on clutch cards. Gathering enough nest records can also help mitigate issues with bias and missing information. Ultimately, museum collections are beneficial for long-term phenological analyses because researchers can obtain and utilize valuable information from the collections to answer important scientific questions over a long span of time and space [44,57,58].

## 5. Conclusions

Our findings demonstrate that different species are affected in varying ways by their environments. Stilt laying dates became later at higher temperatures and at more inland locations. Willet laying dates became earlier at higher temperatures, as predicted, and while this trend was also present for plovers, latitude was the most important factor for this species. The variability we and previous studies have found among species’ responses to changes in climate makes it even more important for additional studies on shorebird nesting phenology to be conducted. To determine the best conservation practices that should be implemented for shorebirds, we need a better understanding of exactly how these species might be influenced by changes in climate, especially considering how vulnerable they likely are to such changes [22].

## Figures and Tables

**Table 1:** Summary of metrics used to determine laying date for each species.

Species	Average Clutch Size	Average Incubation Period	Assumed Laying Period
Stilt	4	25	4
Willet	4	26	6
Plover	3	25	5

**Table 2:** Number of clutches for each species for each source used in this study. Records for sources with asterisks (*) were retrieved from the Arctos Database website (https://arctos.db.org, accessed on October 2021).

Source for Clutch	Stilt	Willet	Plover	Total
American Museum of Natural History [37]	0	20	27	47
Clemson University Bob and Betsy Campbell Natural History Museum	7	36	26	69
Denver Museum of Nature and Science * [38]	15	5	6	26
Jurica-Suchy Nature Museum at Benedictine University * [39]	1	1	4	6
Museum of Vertebrate Zoology * [40]	27	2	9	38
Smithsonian National Museum of Natural History [41]	5	9	27	41
Western Foundation of Vertebrate Zoology [42]	314	203	181	698
Cornell NestWatch Program	43	11	15	69
Total	412	287	295	994

**Table 3:** Actual terms used by collectors to describe incubation stages of clutches at collection. Similar terms used by collectors were combined into our own incubation stage categories based on the estimated percentage of incubation the clutch had undergone at collection. This percentage was translated into an estimated number of days the eggs of a clutch had likely been incubated based on the typical incubation period for each species. The assumed incubation period for the stilt and plover was 25 days and 26 days for the willet.

Collector Incubation Stage Terms	Incubation Stage Category	Estimated % of Incubation at Collection	Estimated Number of Days of Incubation
All fresh, Fresh, Nearly fresh, Perfectly fresh	Fresh	0%	0
Fresh to slight	Median of “Fresh” and “Slight”	8%	Only applies to two willet clutches: 2
Slight, Slightly incubated, Very slight, Began, Begun, Begun in all, Just begun, Commenced, Barely commenced, Just commenced, Trace, Trace only, Small embryos, Started, Just started, Few days	Slight Incubation	15–16%	4
1/4 incubated, 25%, About 25%	1/4 incubation	25%	Stilt and plover: 6, willet: 7
1/3, 1/3 incubated, 1/3 advanced, About 1/3, About 1/3 advanced, About one third	1/3 incubation	33%	Stilt and plover: 8, willet: 9
2/5 incubated	2/5 incubation	40%	Only applies to one stilt clutch: 10
1/2, 1/2 incubated, Halfway, 1/2 advanced, About 1/2, About 1/2 advanced, Half, About half, About one half, Medium embryos, Well along, Well begun, Well started, 50% incubated, Medium, Nearly 1/2	1/2 incubation	50%	13
Advanced, Advanced-far, Highly advanced, Large embryos, Heavy, Considerable, Far advanced, Very far advanced, Well advanced	Advanced Incubation	80%	Stilt and plover: 20, willet: 21
Ready to hatch, Nearly ready to hatch, Feathers ready to hatch	Two days left of incubation	96%	Stilt and plover: 24, willet: 25

**Table 4:** Summary of the linear model results for our first analysis investigating if CID for all species combined has changed over time or space. Results reported are only for the best model and only include significant effects and interactions. Total nests = 733, stilt = 339, willet = 198, and plover = 196 for nests from 1852–1989.

Fixed Effects	Estimate	*p*-Value
Nest Year	−0.09	0.003
Nest Latitude	1.75	0.008
Nest Longitude	2.03	<0.001
Species: Stilt	77.34	<0.001
Species: Willet	−54.91	<0.001
Species: Plover	−22.43	0.03
Species (Stilt) × Longitude	2.39	0.02
Species (Willet) × Longitude	−0.57	0.37
Species (Plover) × Longitude	−1.82	0.02

**Table 5:** Summary of the mixed linear model results for our analysis investigating if temperature (**A**) or precipitation (**B**) in our study areas in the west (for stilt nests) and east (for plover and willet nests) have changed over time and space for the first four months of the breeding season. In these models, the weather station ID where temperature and precipitation data were retrieved was always treated as a random effect to control for multiple samples taken over the years the station was active. Results reported are only for the best models (effects with dashed lines were insignificant and removed from the final model). Climate data were retrieved from 239 stations in California close to the stilt nests and 432 stations in the east (Florida, Georgia, North Carolina, South Carolina, Virginia) close to the plover and willet nests from 1851–2022.

A		West Temperature			East Temperature		
Month	Fixed Effects	Adjusted R^2^	Estimate	*p*-Value	Adjusted R^2^	Estimate	*p*-Value
March	Year	0.83	0.02	<0.001	0.80	0.004	0.02
	Latitude		−1.17	<0.001		−1.11	<0.001
	Longitude		-	-		-	-
April	Year	0.84	0.01	<0.001	0.86	0.01	<0.001
	Latitude		-	-		−0.84	<0.001
	Longitude		0.90	<0.001		-	-
May	Year	0.87	0.01	<0.001	0.80	0.004	<0.001
	Latitude		-	-		−0.43	<0.001
	Longitude		0.88	<0.001		−0.32	<0.001
	Year	0.92	0.01	<0.001	0.69	0.01	<0.001
June	Latitude		0.96	0.01		−0.22	<0.001
	Longitude		1.68	<0.001		−0.24	0.001
**B**		**West Precipitation**			**East Precipitation**		
**Month**	**Fixed Effects**	**Adjusted R^2^**	**Estimate**	***p*** **-value**	**Adjusted R^2^**	**Estimate**	***p*** **-value**
March	Year	0.28	-	-	0.09	−0.14	<0.001
	Latitude		−4.88	0.006		6.76	<0.001
	Longitude		−13.15	<0.001		−6.68	<0.001
April	Year	0.23	-	-	0.09	0.21	<0.001
	Latitude		−1.76	0.053 ^1^		3.66	<0.001
	Longitude		−4.57	<0.001		−4.08	<0.001
May	Year	0.14	-	-	0.07	-	-
	Latitude		1.68	<0.001		−4.71	<0.001
	Longitude		-	-		7.02	<0.001
	Year	0.15	-	-	0.20	0.28	<0.001
June	Latitude		1.12	<0.001		−9.71	<0.001
	Longitude		0.41	0.04		-	-

^1^ This model had the lowest AIC score even though latitude is only near significant.

**Table 6:** Summary of the linear model results for our analysis investigating if nest temperature (A) or nest precipitation (B) could significantly predict clutch initiation date (CID) for the stilt, willet, and plover, and which monthly average was the best predictor. In this model, the only fixed effect was either nest temperature or nest precipitation.

A	Species	Month	R^2^	*F*-Value with df	Estimate	*p*-Value	n
	Stilt	March	0.01	2.28 _1, 210_	0.85	0.13	212
		April	0.01	3.00 _1, 211_	0.93	0.08	213
		May	0.02	5.02 _1, 212_	0.81	0.03	214
		June ^1^	0.04	8.52 _1, 219_	0.86	0.004	221
	Willet	March	0.08	9.22 _1, 113_	−2.10	0.003	115
		April	0.13	16.10 _1, 111_	−2.93	<0.001	113
		May ^1^	0.15	20.11 _1, 110_	−4.07	<0.001	112
		June	0.07	8.71 _1, 108_	−3.96	0.004	110
	Plover	March	0.01	0.84 _1, 75_	−0.81	0.36	77
		April ^1^	0.0233	1.83 _1, 77_	−1.17	0.18	79
		May	0.0227	1.80 _1, 77_	1.95	0.18	79
		June	0.017	1.33 _1, 75_	2.09	0.25	77
**B**	**Species**	**Month**	**R^2^**	***F*-value with df**	**Estimate**	***p*-value**	**n**
	Stilt	March ^1^	0.014	3.12 _1, 221_	−0.07	0.08	223
		April	0	0.15 _1, 215_	−0.02	0.70	217
		May	0	0.01 _1, 207_	−0.01	0.93	209
		June	0.011	2.32 _1, 213_	−0.38	0.13	215
	Willet	March	0	0.15 _1, 124_	0.01	0.70	126
		April	0.017	2.05 _1, 118_	0.04	0.15	120
		May	0	0.08 _1, 126_	0.01	0.78	128
		June ^1^	0.018	2.35 _1, 125_	−0.03	0.13	127
	Plover	March	0.01	0.60 _1, 95_	0.02	0.44	97
		April	0	0.03 _1, 93_	−0.01	0.87	95
		May ^1^	0.03	2.75 _1, 96_	−0.05	0.10	98
		June	0.01	1.05 _1, 96_	−0.02	0.31	98

^1^ This model had the highest R^2^ and was considered to be the best predictor of CID for that species.

## Data Availability

Data is contained within the article or Supplementary Materials. The datasets used in this study are available in Supplementary Table S1, File S1 and File S2.

## Supplementary Materials

The following supporting information can be downloaded at: https://www.mdpi.com/article/10.3390/ani13152459/s1, Table S1: Records of all Nests with Justification for Estimated CID; File S1: Temperature and Precipitation Tables for the East and West Regions; File S2: CID vs. Temperature and Precipitation Tables for all Three Species.
